# Can Exercise-Mediated Adipose Browning Provide an Alternative Explanation for the Obesity Paradox?

**DOI:** 10.3390/ijms26051790

**Published:** 2025-02-20

**Authors:** Jiani Zhao, Xuehan Li, Chunyu Liang, Yi Yan

**Affiliations:** 1 Department of Sport Biochemistry, School of Sport Science, Beijing Sport University (BSU), Beijing 100084, China; zjn@bsu.edu.cn (J.Z.); 2020110078@bsu.edu.cn (X.L.); 2 School of Physical Education, Guangxi University (GXU), Nanning 530004, China; 3 Laboratory of Sports Stress and Adaptation of General Administration of Sport, Beijing Sport University (BSU), Beijing 100084, China; 4 Exercise and Physical Fitness, Beijing Sport University (BSU), Beijing 100084, China

**Keywords:** obesity paradox, cardiovascular disease, adipose browning, exercise

## Abstract

Overweight patients with cardiovascular disease (CVD) tend to survive longer than normal-weight patients, a phenomenon known as the “obesity paradox”. The phenotypic characteristics of adipose distribution in these patients (who survive longer) often reveal a larger proportion of subcutaneous white adipose tissue (scWAT), suggesting that the presence of scWAT is negatively associated with all-cause mortality and that scWAT appears to provide protective benefits in patients facing unhealthy states. Exercise-mediated browning is a crucial aspect of the benign remodeling process of adipose tissue (AT). Reduced accumulation, reduced inflammation, and associated adipokine secretion are directly related to the reduction in CVD mortality. This paper summarized the pathogenetic factors associated with AT accumulation in patients with CVD and analyzed the possible role and pathway of exercise-mediated adipose browning in reducing the risk of CVD and CVD-related mortality. It is suggested that exercise-mediated browning may provide a new perspective on the “obesity paradox”; that is, overweight CVD patients who have more scWAT may gain greater cardiovascular health benefits through exercise.

## 1. Introduction

Cardiovascular disease (CVD) continues to be the primary cause of mortality on a global scale, significantly affecting both the quality of life of the inflicted and healthcare expenses [1]. CVD was identified as the cause of an estimated 20.5 million deaths worldwide in the year 2021 [2]. Traditionally, body mass index (BMI) in individuals with obesity has been positively correlated with adipose tissue (AT) content, cardiovascular risk, and mortality [3]. For example, a study that followed 5881 participants for up to 14 years found a dose-dependent relationship between BMI and the risk of developing heart failure (HF), with each one-unit increase in BMI associated with a heightened risk of HF, within a certain range [4]. However, emerging evidence reveals a paradoxical relationship between BMI and outcomes in CVD: overweight patients with CVD often demonstrate improved survival rates compared to their normal-weight counterparts, a phenomenon termed the “obesity paradox” [5,6,7]. A study following 1203 patients with advanced systolic HF showed that, among patients who did not undergo transplantation, those with lower BMI had the highest mortality, followed by normal-weight HF patients. In contrast, patients with higher BMI had lower all-cause mortality [5]. Surprisingly, these patients with high BMI and low mortality often showed a different pattern of AT distribution than patients with low BMI and high mortality: less visceral adipose tissue (vWAT) and more subcutaneous white adipose tissue (scWAT).

The differences among dynamic distribution and endocrine function with differing AT content play a role of great importance in metabolism regulation. There is growing evidence that changes in the basic morphology and physiological and metabolic activity of AT affect the health of patients with CVD. Disequilibrium or dysfunctional AT lead to obesity and metabolic disorders, which in turn are risk factors for cardiovascular complications [8]. As major AT depots, vWAT and scWAT are believed to have distinct metabolic roles. A review of human observational studies from multiple databases (from January 2000 up to October 2023), using the vWAT-to-scWAT ratio as an independent variable and CVD as an outcome variable, found that higher vWAT-to-scWAT ratios were significantly associated with increased risk of CVD [9].

Different from white adipose tissue (WAT), which is mainly responsible for energy storage and buffering, there is also brown adipose tissue (BAT), which is mainly responsible for thermogenesis, and beige AT, between WAT and BAT. BAT has a positive effect on many metabolic diseases, including CVD [10]. A large-scale study of 53,475 patients found that patients with detected brown fat had a 32 percent lower risk of coronary artery disease, a 23 percent lower risk of cerebrovascular disease, a 38 percent lower risk of congestive HF, and a 15 percent lower risk of hypertension [11]. However, BAT is present in rodents throughout life. In humans, BAT is found primarily in infants and young children, with very low levels in adults [12]. Research shows when the sympathetic nerves are excited, such as when the body is in a state of hypothermia or exercise, the WAT is transformed into a BAT phenotype; that is, WAT browning (or beige-ing) occurs [13,14,15,16,17,18]. Given the widespread distribution and abundance of white AT in the body, promoting browning is a potential novel way to improve CVD and related disease [19,20,21].

**Figure 1:**
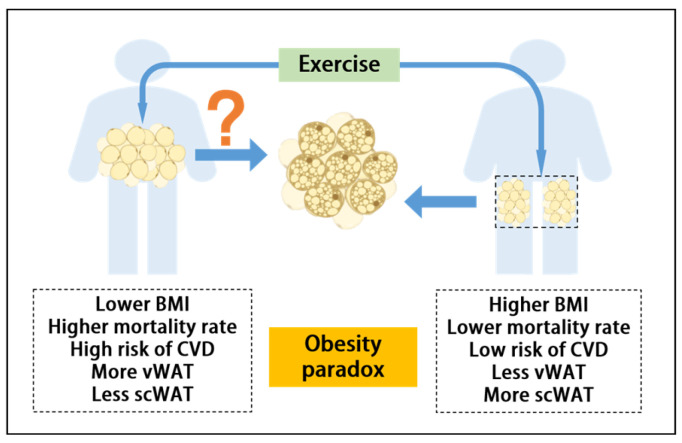
An important aspect of exercise in promoting cardiovascular health is that exercise promotes browning of WAT, and exercise promotes browning of scWAT more than vWAT. At the heart of the “obesity paradox” is that people with high BMI have higher scWAT and lower vWAT, resulting in a healthier phenotype. Therefore, exercise further promotes health by promoting the browning of scWAT in this population.

Exercise is not only recognized as an effective way to reduce CVD mortality [21,22], but also an effective means of promoting the healthy remodeling of AT, one of which is browning. In particular, scWAT not only has a certain protective effect against CVD but is also more sensitive to exercise-mediated browning than vWAT, so it may be an entry point to explain the obesity paradox in exercise [23,24]. In this review, we discussed the phenotypic and metabolic characteristics of AT in patients with CVD. Based on the relationship between exercise-mediated browning and healthy obesity phenotype in patients with CVD and its potential health benefits, it was hypothesized that individuals with obesity might have a higher potential for exercise-mediated browning, which could partially explain the obesity paradox (Figure 1).

## 2. The Relationship Between AT and CVD

AT is a complex organ integral to cardiovascular physiological regulation. As a metabolic storage tissue, it primarily serves to store lipids and maintain energy balance. Furthermore, it possesses endocrine functions, thermogenic properties, and heat preservation effects. Recent studies have demonstrated that AT can engage in the regulation and information exchange processes of whole-body metabolic health through the release and reception of metabolic factors [25]. The metabolic characteristics of AT exhibit a close correlation with its anatomical location (e.g., scWAT and vWAT [26]) and the metabolic signaling molecules intimately associated with it (including inflammatory mediators [27,28] and adipokines [25]). These factors are pivotal in the pathogenesis of CVD. Consequently, this discussion will delve into the potential underlying link between AT and CVD through three dimensions: AT distribution, inflammatory response, and adipokines.

### 2.1. AT Distribution and Its Impact on CVD

The emergence of the “obesity paradox” has triggered new thinking about whether the buildup of AT is beneficial to some extent for some lesions, and whether the buildup of AT is protective against disease progression in patients with CVD. A study was performed that analyzed 2710 subjects with magnetic resonance imaging (MRI) and found that the distribution of vWAT was higher in those with central adiposity, which induced concentric left ventricle remodeling and adverse hemodynamics, and that the greater the distribution of scWAT in individuals with the same volume of adiposity, the more likely it seemed to be to obtain a healthy body phenotype [29]. Emerging evidence also suggests that ectopic deposition of vWAT, including liver and epicardium, may increase atherosclerosis and cardiometabolic risk [30]; that is, vWAT has a greater cardiometabolic risk than scWAT [31]. Interestingly, vWAT accounts for only 5–20% of total body fat volume but induces a much higher risk of CVD [32]. BMI is therefore not a complete indicator of cardiometabolic disease risk because it does not reflect individual differences in AT deposition [33]. It has been supported by the findings that fasting glucose levels, triglycerides, and complication burden were higher in vVAT patients than in scWAT patients, even after accounting for BMI [34]. Recently, the International Atherosclerosis Society and the Chair of the International Cardiometabolic Risk Task Force on Visceral Obesity jointly issued a statement supporting vWAT as an independent risk factor for cardiovascular morbidity and mortality, while BMI does not determine cardiovascular risk [35], suggesting that AT distribution may be a more important indicator of CVD than total AT volume. The reason why the obesity paradox exists is precisely because the AT of these people with obesity is mostly concentrated in the subcutaneous rather than the visceral area; that is, healthy obesity. However, AT e in patients with CVD is more concentrated in the visceral area, which causes the probability of CVD and its mortality rate to be lower in people with relatively large BMI.

The distribution of AT, particularly the differences between scWAT and vWAT, plays a pivotal role in determining their metabolic and cardiovascular effects. Compared to adipocytes in other parts, subcutaneous adipocytes have distinctly different gene expression patterns (higher expression of lipocalin, lower expression of pro-inflammatory adipokines) [36]. Studies of human adipocyte differentiation patterns in vitro have found that subcutaneous adipocyte precursors have a higher differentiation and lipogenic potential. In contrast, there is evidence that human subcutaneous adipocytes have lower lipogenic potential than AT in other locations, and studies have shown that intermittent fasting can significantly reduce harmful vWAT but not scWAT, revealing that scWAT has greater plasticity in different physiological states [37,38,39]. In insulin-resistant mice, a prolonged high-fat diet leads to AT expansion and scWAT has a lower proliferative capacity and expands mainly by accumulating more triglycerides, whereas vWAT expands by both proliferation (increasing cell number through preadipocyte differentiation) and hypertrophy (increasing cell size through lipid droplet expansion) [40,41]. Although the results of these studies are inconsistent, they all show that scWAT and vWAT possess different abilities and phenotypes in lipid formation and secretion [42]. It is precisely because of these differences that the accumulation of vWAT is more harmful to cardiovascular health.

### 2.2. AT Inflammation and CVD Risk

The “obesity paradox” may involve inflammation [43]. Weight gain leads to an increase in the number of macrophages in AT, which can increase from 10% to more than 40% of the original population [44], while AT undergoes malignant remodeling, as evidenced by the induction of an increase in inflammatory cell lineages, such as macrophages, and a decrease in capillarization of adipocytes, thus limiting nutrient delivery and causing AT fibrosis [45,46]. Inflammation of AT will further lead to increased levels of circulating inflammatory factors [47], thus inducing CVD. Previous studies have found that transplantation of inflammatory AT into the perivascular area of a normal carotid artery can induce atherosclerotic lesions and increase inflammation [48,49], which once again confirms the important role of AT inflammation in the process of CVD.

vWAT is pro-inflammatory, metabolically active, and easy to lipolysis [31], while human scWAT contains more adipocytes, less infiltration of CD68^+^ and M1-activated cells, and expresses higher levels of cardioprotective adipofactors, including adiponectin and irisin [50]. The expansion of vWAT led to the increase of pro-inflammatory M1 macrophages, whereas the increase of scWAT led to the increase of anti-inflammatory M2 macrophages [51]. Therefore, vWAT inflammation is the main culprit leading to systemic inflammation and even CVD [31,52]. Obesity-induced AT accumulation promotes the up-regulation of pro-inflammatory adipokines and the down-regulation of anti-inflammatory adipokines, which have a direct effect on the cardiovascular system and indirectly contribute to CVD through metabolic alterations in the liver, skeletal muscle, and heart [45]. For example, chronic systemic inflammation of obesity further promotes the accumulation of epicardial fat and adversely disrupts the development of epicardial fat to pro-inflammatory phenotypes [53,54]. Pro-inflammatory adipokines and lipids secreted by epicardial AT can affect cardiomyocytes and the extracellular matrix through paracrine mode [55], thereby mediating the effects of systemic inflammation on neighboring myocardium [56]. In addition, damaged cardiomyocytes can also release pro-inflammatory cytokines such as IL-6 and TNF-α, triggering the lipidation of epicardial AT, leading to cardiac cachexia and worsening prognosis [57], thus forming a vicious circle in HF patients. This shows that obesity leads to chronic inflammation of AT, and chronic inflammation aggravates obesity, thus promoting the occurrence and development of CVD.

### 2.3. AT Adipokines and CVD Risk

Adipokines are a variety of bioactive compounds mainly secreted by AT, which act on the systemic vascular system through endocrine/paracrine mode, acting on energy balance, immune response, vascular homeostasis, angiogenesis, insulin sensitivity, and lipid metabolism, and ultimately affect CVD directly or indirectly through these effects [58,59]. Studies have shown an association between adipokines and CVD.

The expression and secretion of many adipokines, such as leptin, resistin, tumor necrosis factor-α (TNF-α), interleukin-6 (IL-6), C-C motif chemokine ligand 2 (CCL2), CXC motif chemokine ligand 5 (CXCL5), and angiopoietin-like 2, are up-regulated in AT during obesity. Additionally, it acts as a pro-inflammatory factor to promote obesity-related metabolic diseases and CVDs [59,60,61,62]. Research found that leptin can induce inflammation and endothelial dysfunction, cause vasoconstriction, and promote the migration and proliferation of vascular smooth muscle cells, increasing the vulnerability and rupture risk of atherosclerotic plaques [63]. On the other hand, adipocytes also secrete a few anti-inflammatory adipokines with protective effects, such as adiponectin, secreted frizzled-related protein 5 (sFRP5), C1q, tumor necrosis factor-associated protein 9, and omentin [64,65,66,67]. Dysfunction of anti-inflammatory adipokines disrupts metabolic homeostasis, leading to CVD. Studies have shown the high adiponectin levels can reduce the risk of coronary artery disease and can be used as a predictor of clinical prognosis in patients with coronary heart disease [68].

Therefore, an in-depth exploration of adipokines may be helpful in the study of obesity-related CVD. Exercise can affect the development of CVD by regulating the secretion and metabolism of adipokines. The following summarizes some of the adipokines associated with CVD (Table 1).

## 3. Exercise-Mediated Browning: A New Avenue for CVD Protection

Exercise is an important means to prevent and treat CVD. A prospective study of the China Kadoorie Biobank (CKB), which followed more than 487,000 individuals without baseline CVD for up to 7.5 years, showed that average daily total physical activity levels were significantly negatively associated with CVD-related mortality [107]. Researchers are progressively exploring the mechanisms underlying the improvement of CVD through exercise. The alterations in AT responses induced by exercise training, encompassing tissue remodeling, increased angiogenesis, and enhanced mitochondrial biogenesis [108], collectively suggest that exercise-induced “browning” of WAT could serve as a pivotal means by which exercise ameliorates CVD by fostering AT health. Additionally, accumulating evidence from both human and animal studies indicates that exercise can direct the metabolic characteristics of WAT towards a more active “browning” phenotype [14,19,20,21]. This further implies that exercise-induced WAT browning may represent a potential therapeutic target for CVD.

### 3.1. Exercise-Mediated Browning Regulates AT Distribution

The specific type of AT that accumulates in the body is critical to health risks. vWAT accumulation is associated with insulin resistance, increased risk of type 2 diabetes, dyslipidemia, atherosclerosis progression, and mortality [109,110,111], while scWAT accumulation is associated with improved insulin sensitivity and reduced risk of type 2 diabetes [112,113].

The obesity paradox suggests that, unlike vWAT, scWAT has a protective effect on CDV, indicating that different fat pools have different regulatory effects on metabolism. For example, recent studies have found that the promotion effect of exercise on the browning of WAT also has the heterogeneity of the AT pool. Exercise can reduce the adipocyte area in scWAT and vWAT and promote the increase of UCP1 expression in WAT of these two parts, but UCP1 expression in scWAT is more significant than that in vWAT [23]. A one-time intervention of swimming for 90 min stimulated the increase of the expression of UCP1 in iWAT, but no change of the browning marker was observed in vWAT [24]. The mice were subjected to voluntary wheel exercise for 11 days, and the mRNA expressions of browning markers PRDM16 and UCP1 in the scWAT of exercise mice were increased compared with those of quiet mice. The immunofluorescence of UCP1 was also shown to increase in scWAT of exercise mice. Compared with the significant effect of exercise on Ucp1 in scWAT, exercise did not increase the expression of UCP1 mRNA in vWAT [16]. Subsequently, scWAT and vWAT of the sedentary mice and the exercised mice were transplanted, respectively. It was found that the AT of the transplanted exercise mice increased in multilocular cells, and, compared with the sham operation mice, the glucose tolerance of both the sedentary mice and the exercise mice were improved [16]. However, transplanting vWAT from the exercise mice had no effect on the glucose tolerance of the transplanted mice [16]. All of the above studies showed that exercise was more likely to stimulate browning of scWAT than vWAT. More rodent studies have further confirmed that different forms of exercise (active wheel exercise, passive treadmill exercise, swimming, etc.) and different exercise cycles (11–63 days) can cause significant browning changes in scWAT, including the increase of browning markers (UCP1, PRDM16, CIDEA, PGC-1α, PPARγ, COX8b, DIO2, etc.) and the increase of multilocular adipocytes [16,17,18]. The reason for the obesity paradox (people with more scWAT, less vWAT, and a large BMI are healthier than those with less scWAT, more vWAT, and a small BMI) is precisely because exercise stimulates the browning of scWAT more, so scWAT can obtain greater health benefits.

While exercise stimulates browning in scWAT, it also has other health benefits. For example, the morphology of scWAT after browning changes, manifesting as a decrease in adipocyte area and an increase in multicompartment droplets [16]. Continuous aerobic exercise for 8 weeks promoted UCP1 mRNA expression in scWAT and improved glucose tolerance, insulin sensitivity, and VO_2max_ in mice [114]. In addition, numerous studies have shown that exercise can increase mitochondrial activity in rodent and human scWAT [16,115,116,117]. In rodents, mitochondrial activity was increased in scWAT after 11 days of voluntary wheel running [16]. Four to eight weeks of swimming exercise increased the expression of mitochondrial marker PGC-1α in mouse scWAT [115,116]. Eight weeks of treadmill training also increased the expression of PGC-1α in rat scWAT [117]. These studies suggest that exercise training has significant effects on scWAT mitochondrial gene expression and activity. Changes in scWAT mitochondrial gene expression occurred in response to different durations (11 days to 9 weeks) and modes of exercise (voluntary wheel cage running, swimming, treadmill running). In humans, the study also determined that both light and vigorous aerobic exercise increased mitochondrial gene expression in scWAT. Following a 6-month intervention with light exercise (3 h of aerobic exercise per week) in previously sedentary healthy men, genes involved in oxidative phosphorylation were increased in scWAT [118]. A 4-week intensive exercise intervention (1 h, 3 times per week) in sedentary men and women with normal glucose tolerance, impaired glucose tolerance, or type 2 diabetes also significantly increased PGC-1α in scWAT [119]. This is particularly important because these data suggest that exercise can increase mitochondrial gene expression in scWAT in subjects with normal blood glucose and type 2 diabetes, providing more evidence for exercise as a therapeutic tool to improve metabolic health in AT.

In general, compared with vWAT, exercise can promote the browning of scWAT, promote the distribution of vWAT in a healthier form and function through the process of browning, and further increase health benefits.

### 3.2. Exercise-Mediated Browning Reduces Inflammation

The “obesity paradox” exists due to the difference between fat tissue from healthy obesity and unhealthy obesity. If the fat in people with obesity is mainly stored under the skin, it usually does not induce inflammation, and can be called healthy obesity; on the contrary, if fat is mainly stored in the internal organs, it may lead to chronic low-grade inflammation, which can be called unhealthy obesity [120].

There is increasing evidence that WAT browning may be significantly associated with a decrease in the levels of inflammatory factors in WAT [108,121]. For example, HIGD1A inhibits the ox-mtDNA/NLRP3 inflammassome/JNK pathway, reduces ROS levels, and mitigates DNA damage in the process of promoting WAT browning, thereby reducing the inflammatory response [122]. Resistance movement can further produce meteorin-like proteins by inducing PGC-1α homologous PGC-1α4 and promote WAT browning by stimulating IL-4 and IL-13 secretion in eosinophils [123]. Studies in rodents have shown that the metabolic benefits of exercise-mediated WAT browning are dependent on the presence of IL-6 and have no effect on IL-6-KO mice [124,125]. In a mouse model of intravascular injury, macrophages accumulate in perivascular WAT, resulting in browning phenotypes. Specific deletion of PRDM16 (a key regulator of browning) in mouse AT showed that deletion of PRDM16 inhibited the browning of perivascular WAT and exacerbated inflammation and pathological vascular remodeling after injury. Conversely, activation of perivascular WAT browning reduces inflammation and pathological vascular remodeling, thereby improving atherosclerosis [126]. Perivascular WAT has been reported to undergo inflammatory changes in response to vascular injury. Here, we show that vascular injury induces the beiging (BAT-like phenotype change) of PVAT, which fine-tunes inflammatory response and thus vascular remodeling as a protective mechanism. In a mouse model of endovascular injury, macrophages accumulate in PVAT, causing beiging phenotype change. Inhibition of PVAT beige-ing by genetically silencing PRDM16, a key regulator to beiging, exacerbates inflammation and vascular remodeling following injury.

Therefore, during the process of promoting browning in WAT, exercise can enhance the functional properties of fat, including mitochondrial function. These alterations are pivotal in ameliorating the inflammatory response. Concurrently, the modulation of the adipose inflammatory microenvironment is a crucial determinant in regulating cardiovascular function, either directly or indirectly [127,128,129]. On this basis, exercise-mediated AT browning has the potential to ultimately facilitate the improvement of cardiovascular function.

### 3.3. Browning-Associated Adipokines Benefit CVD

It is well known that one of the mechanisms of exercise mediated AT browning is exercise can stimulate the secretion of various exercise factors. In recent years, studies have found that exercise factors are closely related to the occurrence and development of CVD [130]. Exercise can stimulate various organs, including WAT, to secrete irisin, thereby promoting browning of WAT [21,130], and irisin has a protective effect on the development of CVD: serum irisin in disease-free centenarians, young healthy controls and patients with precocious acute myocardial infarction were detected. It was found that the serum level of irisin in healthy centenarians was the highest, followed by young healthy controls, while the serum level in young patients with precocious acute myocardial infarction was significantly lower [131]. This suggests that irisin has a positive effect on promoting browning of WAT and protecting the cardiovascular system. In addition to irisin, there are many exercise factors that promote browning that are beneficial to cardiovascular health. This section summarizes some common adipokines related to CVD and browning (Table 2).

#### 3.3.1. Irisin

Exercise causes up-regulation of irisin in plasma. A study conducted a 3-week free exercise intervention in mice, along with a 10-week endurance exercise intervention in healthy humans, and irisin was significantly elevated in the blood of all subjects 2 weeks post-exercise [18,132]. Although irisin is secreted by muscles, exercise-enhanced irisin elevates UCP1 expression in scWAT, which is the best evidence for browning of scWAT, a process that is also driven by PGC-1. FNDC5 is a precursor protein of irisin, and overexpression of full-length FNDC5 using adenovirus also elevates plasma irisin in mice [18]. Administration of an antagonist of FNDC5 to mice resulted in a decrease in irisin expression in WAT in exercise-experienced mice, followed by an attenuation of exercise-mediated browning of WAT, which in turn impaired weight loss from exercise [133]. Irisin has a similarly positive effect on the maintenance of cardiovascular health, with circulating irisin levels positively correlating with levels of diastolic blood pressure in Chinese patients with newly diagnosed type 2 diabetes without clinical vascular lesions [134]. Similarly, serum irisin concentrations were lower in diabetic patients with concomitant atherosclerosis than in diabetic patients without atherosclerosis, suggesting that circulating irisin has the potential to be used as a diagnostic biomarker for monitoring the progression of CVD in diabetic patients [135,136]. In addition, serum irisin concentration is stable in chronic CVD patients, but serum irisin concentration gradually decreases within 48 h after acute myocardial infarction (MI), suggesting that serum irisin concentration may have an important clinical value [137].

#### 3.3.2. FGF21

Exercise also causes an increase in plasma levels of fibroblast growth factor 21 (FGF21), which increases energy metabolism levels in mice [138]. FGF21 is mainly secreted through the liver, but AT is also an organ for FGF21 secretion. After exercise, elevated FGF21 expression in WAT was accompanied by elevated UCP1 expression [21]. FGF21 has been found to act directly on the cardiovascular system and may serve as an early biomarker for CVD [139]. FGF21 protects the heart by regulating oxidative stress, lipid metabolism, autophagy, and apoptosis [139]. However, excessively high FGF21 levels are harmful in patients with CVD, and the higher the plasma FGF21 levels in patients using statins, the higher the risk of coronary heart disease development [140]. By comparing serum FGF21 concentrations in patients with unstable angina pectoris with those in patients with stable angina pectoris, it was found that unstable angina pectoris was significantly higher than stable angina pectoris patients and healthy controls [141].

#### 3.3.3. Lactate

Lactate is also a classical molecule that promotes browning, and increased lactate in adipocytes can cause elevated NADH/NAD^+^ in the mitochondria and increased cellular redox stress, while increased expression of UCP1, which binds excess H^+^, could cellularly alleviate the redox stress and thus promote thermogenesis in white adipocytes [142,143]. As a signaling molecule, lactate couples with the lactate-specific receptor-G protein-coupled receptor 81 (GPR81) on the surface of adipocytes to activate the transcription and translation of UCP1 mRNA and increase UCP1 expression [144,145]. We are aware that a large amount of lactate is produced in the body during exercise, and at the same time, lactate can serve as a source of energy for the myocardium, regulate cardiac electrophysiological activity, regulate the function of vascular smooth muscle cells (VSMCs0), and promote angiogenesis [146]. For the heart, lactate is an important metabolite for the prevention of CVD by promoting angiogenesis through pro-angiogenic NF-kB/IL-8 signaling in order to ameliorate ischemia and hypoxia [147], which contribute to the maintenance of cardiovascular function in patients with myocardial infarction (MI), ischemic cardiomyopathy, and other CVDs [148,149]. And it has been shown that lactate is an important molecule in the prevention of atherosclerosis. Lactate activates GPR81 and inhibits lipolysis in adipocytes through insulin-induced antilipolysis, thus reducing the concentration of fatty acids in plasma, which is positively correlated with atherosclerosis [150,151]. The production of browning-promoting exercise factors during exercise is also an important molecule in the prevention of CVD. Table 2 is a summary of the effects of transport browning-related exercise factors on CVD.

**Table 2:** Browning-associated adipokines affecting CVD.

	Hematological Factors	Functionality	Relationship with Cardiovascular Disease	Reference
Noninflammatory factor				
1	Irisin PGC-1α	Increases energy expenditure and improves glucose homeostasis	Maintaining cardiovascular health and reducing the risk of atherosclerosis	[18,133,135]
2	FGF21	Regulates blood sugar and increases metabolic levels	Protects the heart, but high concentrations are harmful	[21,138,140,141]
3	Lactate	Energy supply, metabolic boost	Reduces risk of atherosclerosis and improves ischemia	[145,146,147]
4	12,13-diHOME	Increased intake of fatty acids	Regulates calcium circulation, enhances cardiac function, and enhances cardiac hemodynamics	[152,153,154]
5	Bcl2l13	Induction of mitochondrial fracture and mitochondrial autophagy to ameliorate mitochondrial dysfunction	Improvement of HF	[155,156,157]
6	Apelin	Enhancement of mitochondrial function	Decreases in systolic and diastolic blood pressure and increases in blood flow regulate vascular tone, promote vascular development, and maintain cardiomyocyte homeostasis	[158,159,160]
7	BAIBA	Regulation of lipid metabolism	Treatment of metabolic syndrome and its cardiovascular complications and improvement of atherosclerosis	[161,162]
8	METRNL	Regulation of metabolism and inflammation	Associated with metabolic or CVD (type 2 diabetes, coronary heart disease)	[163,164,165,166]
9	Myostatin	Increased energy substrate uptake	Improvement of cardiovascular risk factors	[167,168,169]
10	Follistatin	Promotes energy metabolism, regulates insulin and glucagon	Myocardial injury protective factor	[170,171,172]
11	GABA	Inhibits insulin secretion	Treatment of CVD-related	[162,173]
12	SPARC	Regulates cell function and tissue remodeling	Affects hemodynamics and cardiac function	[174,175]
13	VEGF	Promoting exercise-induced neurogenesis	Promotes angiogenesis	[176,177]
Inflammatory factor				
14	IL-6	Pro-lipolytic, anti-inflammatory, promotes glucose uptake	Improvement of glucose tolerance, affecting cardiometabolic diseases and CVDs	[86,178,179]
15	TNF-α	Induction of involvement in inflammation	Induction of ROS production leads to endothelial dysfunction in many pathophysiologic conditions	[180,181]
16	TGFβ1	Immune cell chemokines that affect skeletal muscle growth	Regulation of cardiorespiratory fitness and remodeling	[182,183]

## 4. Conclusions and Future Perspective

**Figure 2:**
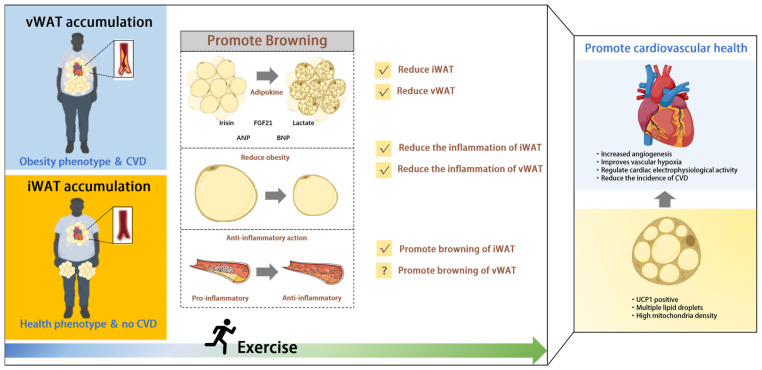
Exercise not only improves cardiovascular health through WAT browning but also reduces fat mass and fat inflammation. The above benefits are more significant in scWAT. This explains the obesity paradox: people with more scWAT, even if they have a higher BMI, gain greater health benefits from exercise-stimulated WAT browning.

The obesity paradox is a theory that has emerged in response to the nonlinear relationship between all-cause mortality and BMI in CVD patients. Although it appears that obese CVD patients are less likely to die, it does not mean that obesity is healthy, and obese patients tend to need more fat reserves than normal-weight people to counteract weight loss in the event of ill health. Current findings indicate the distribution of AT is perhaps what explains the obesity paradox and that healthy phenotypes can be maintained even with a large proportion of scWAT, as long as the amount of vWAT is not extremely expansive over a short period of time. Exercise as a non-drug intervention has been widely proven to have a positive impact on cardiovascular health. This review concluded that exercise can significantly improve risk factors for CVD by promoting browning of WAT. The specific mechanisms may include the changing of WAT distribution, regulating of inflammatory response in WAT, and stimulating an increase in adipokines. Thus, we hypothesize that individuals with obesity might have a higher potential for browning, which could partially explain the obesity paradox and provide a new perspective for the prevention and treatment of CVD (Figure 2).

While exercise-mediated browning offers a promising angle, more experimental and clinical evidence is needed to establish its role in explaining the obesity paradox. (1) On species differences: It has been found in rodents that exercise can promote the browning of WAT through various ways [21]. However, in humans, whether exercise can promote the browning of WAT has been a great controversy. In previous studies [184,185], exercise- mediated WAT browning response was not observed in multiple types of people. However, it has also been demonstrated that 12 weeks of power cycling can increase UCP1 expression in human scWAT [14]. Recent studies have suggested that vWAT may be the main site of the exercise-mediated browning phenotype in humans, different from rodents: browning-related genes are more highly expressed in human vWAT [186], and the browning phenotype was more effective in inducing human vWAT at low temperatures compared with scWAT [15,187]. At present, whether exercise can promote browning of human WAT is controversial, and more evidence is needed in the future to directly support the role of exercise in regulating the browning of human WAT, rather than just rodent experiments. (2) On gender differences: Studies have shown that exercise training is conducive to changing the phenotype of scWAT in male mice, including reducing fat mass, improving mitochondrial function, inducing WAT browning, and stimulating the secretion of adipokines, but these changes do not occur in female mice [188], suggesting that more studies are needed in the future to clarify the gender differences in exercise-promoted WAT browning. (3) On exercise dose: Although moderate-intensity continuous aerobic training (MICT) is the most commonly prescribed intervention, HIIT interspersed with active/passive recovery periods has gained popularity in recent years. HIIT has proven safe in the cardiac rehabilitation setting [189,190], and although there is some debate, meta-analysis evidence suggests that HIIT is more effective, or at least more efficient, than MICT in improving cardiorespiratory fitness and quality of life in patients with CVD [191,192,193]. The same is true in promoting the browning of WAT. The role of resistance training (RT) should not be disregarded, as this modality has proven feasible and effective for most CVD and the promotion of browning [194,195]. Although various exercises can stimulate browning and improve CVD, whether there is an optimal exercise dose remains to be further investigated. (4) On CVD reverse-regulation WAT: For example, we tend to assume that obesity is the cause of CVD in CVD patients with concomitant obesity phenotypes that we see during research, but is there an unhealthy state brought on by CVD that leads to a buildup of AT in patients to cope with emergencies? This hypothesis would break down the concept of preventing CVD through exercise for weight loss and shift to exercise for treating AT accumulation brought on by CVD, thus relieving CVD stress. In conclusion, exercise-mediated browning as an explanatory mechanism for the obesity paradox remains hypothetical and needs further experimental and clinical data to support it.

## Figures and Tables

**Table 1:** Adipokines associated with CVD.

	Adipokines	Function in CVD	Reference
Beneficial			
1	Omentin-1	Reduces inflammatory response from TNF-α	[69,70,71]
2	Adiponectin	Protects cardiovascular smooth muscle and inhibits cholesterol uptake by LDL receptor	[72,73,74]
3	SFRP5	Reduces atherosclerosis	[71,75]
4	Cardiotrophin-1	Maintains blood cholesterol levels	[71,76]
5	VEGF	Promotes angiogenesis	[77,78]
6	Nesfatin-1	Prevention of ischemia-reperfusion injury and regulation of arterial blood pressure	[79,80]
7	FSTL1	Prevention of ventricular hypertrophy, cardiomyocyte apoptosis, and promotion of proliferation	[81,82]
8	CTRPs	CTRP-1 and CTRP-3 prevent coronary artery disease and ischemic injury	[81,83]
9	WISP1	Promotes myocardial repair and prevents apoptosis	[81,84]
Pernicious			
10	Resistin	Promotes coronary artery calcification	[85]
11	TNF-α	Diminished vascular elasticity, endothelial cell apoptosis, impaired myocardial function	[86]
12	FABP-4	Promotes hypertension, atherosclerosis, impairs myocardial contraction	[71,87]
13	Asprosin	Increased triglyceride levels	[71,88]
14	RBP4	Pro-inflammation and ventricular hypertrophy	[89,90]
15	Lipocalin-2	Pro-inflammatory, positively associated with atherosclerosis, myocardial infarction	[81,91]
16	Chemerin	Pro-atherosclerotic, positively correlated with cholesterol levels	[71,81,92]
17	Visfatin	Atherosclerosis, myocardial infarction	[93,94]
18	Apelin	Causes high blood pressure, HF, and impairs myocardial contraction	[95]
19	Gremlin-1	Promotes macrophage migration	[96,97]
20	SAA3	Predictive marker of CVD, up-regulation of TNF-α	[93,98]
21	FAM19A5	Inhibition of angiogenesis	[99]
Lack or too much is harmful			
22	Leptin	Increases macrophages, promotes atherosclerosis, controls blood pressure	[100,101]
23	Interleukin	IL-1, causes hypotension IL-6, associated with atherosclerosis IL-4/13, promotes M2 macrophage recruitment	[86,90,102,103]
24	Vaspin	Pro-atherosclerosis; prevention of vascular endothelial cell apoptosis	[81]
25	PAI-1	Reduction of myocardial fibrosis	[93,104]
26	SPARC	Promotes myocardial injury and fibrosis	[81,105]
27	PGRN	Anti-inflammatory by lowering TNF and promoting myocardial repair after ischemia	[81,106]
